# Being Guided by Your Brain or by Your Heart? Challenges in Adaptive Deep Brain Stimulation

**DOI:** 10.1002/mdc3.70337

**Published:** 2025-08-28

**Authors:** Mario Sousa, Gerd Tinkhauser

**Affiliations:** ^1^ Department of Neurology Bern University Hospital and University of Bern Bern Switzerland

**Keywords:** adaptive deep brain stimulation (DBS), Parkinson's disease, cardiac artifacts

A recurring and fundamental question in life, whether to follow the heart or the brain, is now becoming a real consideration for movement disorders patients undergoing novel and personalized treatment regimes. Adaptive deep brain stimulation (aDBS) adjusts stimulation in real time based on neurophysiological biomarkers and represents a major technical advancement in translating decades of neuroscience research into patient benefit.^1^, ^2^ We are at the very beginning of this new era, and anticipated and unforeseen challenges start to be uncovered.^3^ One fundamental prerequisite for aDBS is the identification of an optimal feedback signal that reliably reflects symptom and medication states.^4^, ^5^ Another essential need is robust technology to minimize confounding factors that could affect algorithm accuracy.^6^, ^7^, ^8^

We report a 41‐year‐old man with Parkinson's disease who underwent implantation of a Medtronic Percept PC stimulator (Minneapolis, MN) and SenSight (B33005) leads. The neurostimulator was implanted in the left chest due to the patient's lifestyle preference to minimize interference with his right‐handed forehand in tennis. Following observations were made during the neurophysiological assessment: as feedback signal, we selected the bilaterally present 11.72‐Hz β peak ±2.5 Hz (Fig. 1A). Video 1, segment 1 shows indefinite streaming in single‐threshold mode with the manufacturer's preset parameters (average window duration [AWD]: 0.1 s). The local field potential (LFP) trace is characterized by rhythmic and regular peaks occurring at 89/min. When the patient raises his arms, the amplitude of these peaks decreases, and increases again when the arms are lowered. Subsequently (segment 2), the patient is asked to perform a modified version of the Valsalva maneuver (deep inhalation followed by breath holding) resulting in a decrease in both amplitude and frequency of the recorded peaks, with recovery upon resumption of normal breathing. Segment 3 shows the dual‐threshold mode, where similar signal fluctuations were inducible by the arms‐up maneuver, though now at a slower temporal scale due to increased signal smoothing of the preset configurations (AWD: 1.2 s). Video 2 demonstrates the single‐threshold mode where DBS was systematically triggered by these repetitive peaks, even though not perfectly matching each peak due to the default blanking time after a triggered burst of stimulation. It is also possible to depict the effect of this modified Valsalva maneuver in the aDBS response. Video 3 demonstrates the dual threshold with aDBS activated, showing a slow LFP amplitude decrease over time, with arms raised that leads to a decrease in stimulation amplitude.

**FIG. 1:**
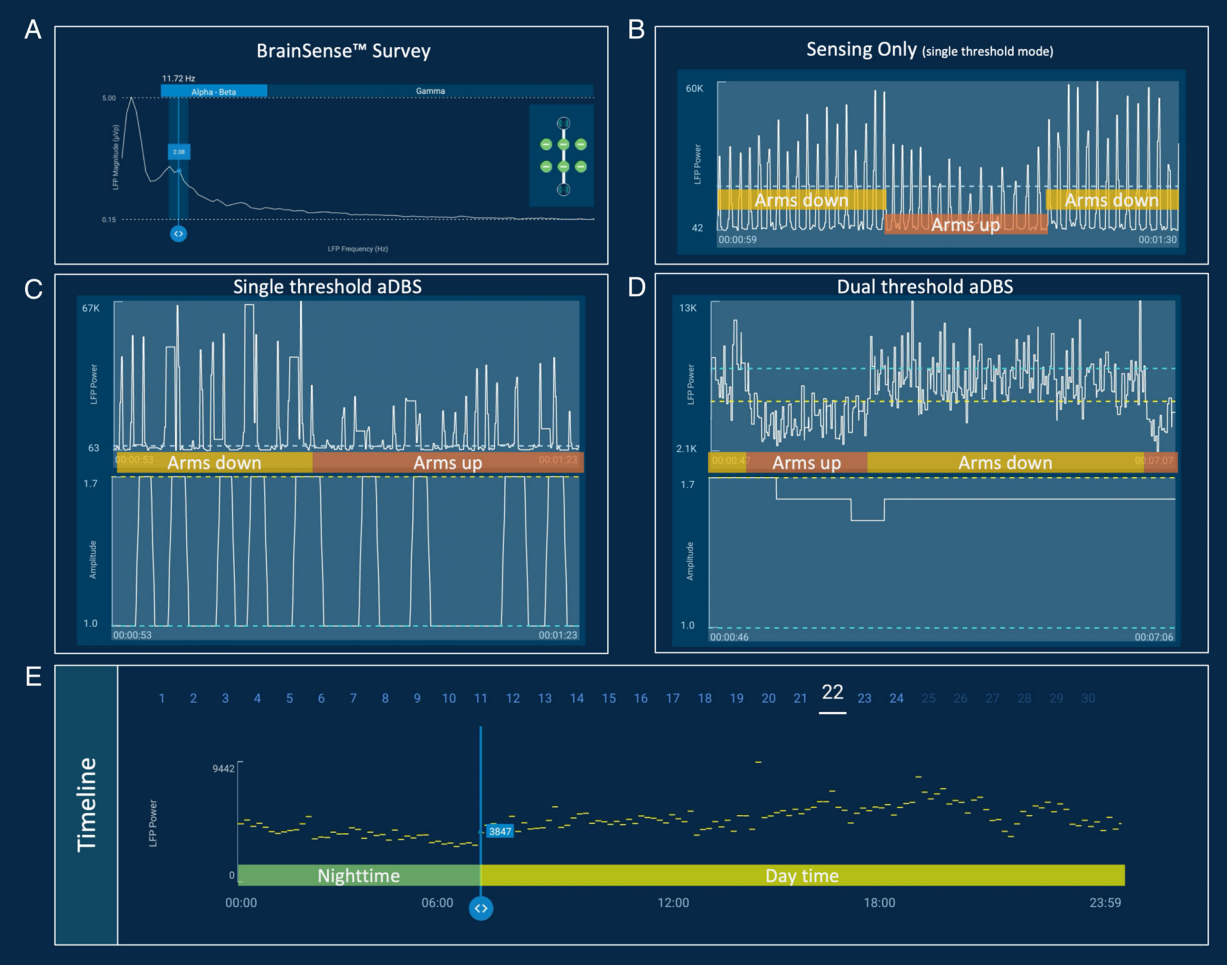
Workflow to detect cardiac artifacts during aDBS (adaptive deep brain stimulation) setup. (**A**) β‐Peak selection; (**B**) “arms‐up” maneuver for detecting cardiac artifacts; (**C**) heart‐driven stimulation changes in single‐threshold mode and (**D**) dual‐threshold adaptive mode; (**E**) circadian timeline dynamics. No offline signal‐processing applied.

**Video 1:** Cardiac artifact detection during aDBS (adaptive deep brain stimulation) setup. Effects of the arms‐up (segment 1) and modified Valsalva (segment 2) maneuvers in sensing‐only single‐threshold mode; dual‐threshold mode shows similar artifact attenuation with slower dynamics due to increased signal averaging (segment 3).

**Video 2:** Heart‐driven aDBS (adaptive deep brain stimulation) in single‐threshold mode. Cardiac artifacts repeatedly triggering stimulation. The modified Valsalva maneuver reduces LFP amplitude and modifies the aDBS response.

**Video 3:** Heart‐driven aDBS (adaptive deep brain stimulation) in dual‐threshold mode. The arms‐up maneuver reveals that stimulation was triggered by cardiac artifacts rather than by the intended brain‐derived β‐activity biomarker.

The heart acts as a strong dipole, and the aforementioned observations are consistent with electrocardiogram (ECG) contamination of the brain signals.^9^ Raising the arms or inhaling increases the distance between the neurostimulator and cardiac dipole, which can reduce artifacts. The modified Valsalva maneuver also lowers artifact amplitude by weakening the heart's electrical field due to the reduced venous return and stroke volume.

Left‐sided neurostimulator placement carries a higher risk of ECG artifacts, so right‐sided implantation is generally recommended. Nonetheless, artifacts can still occur with right‐sided devices and may be absent on the left.^9^ Implantation decisions should consider patient lifestyle, anatomy, and nowadays informed counseling on sensing implications. Although future solutions such as real‐time artifact removal, optimized montages, or skull‐mounted implantable pulse generators (IPGs) are promising, for now vigilance and practical detection strategies remain essential.

We propose a quick, clinic‐friendly method to ensure aDBS is truly brain driven. We primarily suggest performing this screening using the single‐threshold mode due to its higher temporal resolution. The screening includes (1) visible inspection of the LFP trace for repetitive peaks matching heart rate with the patient at rest (Fig. 1B); (2) arms‐up maneuver, asking the patient to raise both arms and observe whether the LFP trace changes in amplitude or morphology (Fig. 1C,D); and optionally (3) a modified Valsalva maneuver, asking the patient to inhale and hold the breath and again observe for changes in amplitude or signal morphology of the LFP trace. This option however is not feasible for all patients. Note, the arms‐up maneuver could also be performed in the dual‐threshold mode (Fig. 1D), yet the longer smoothing time constants may visually mask ECG contamination and amplitude changes occur at a much slower time rate. Importantly, the long‐term recording mode (timeline, Fig. 1E), although useful for adjusting aDBS thresholds, may not reliably detect ECG artifacts in LFP signals, as both LFP power and cardiac parameters can follow similar patterns: both decrease during nighttime and increase during daytime, and both are augmented by physical activity.^4^ Conceptualizing future clinical‐neurophysiological strategies that integrate both in‐hospital and ambulatory assessments may therefore be critical to evaluate the impact of biological artifacts, determine individually optimal feedback frequencies, and calibrate aDBS.^2^, ^10^

In conclusion, the degree to which ECG artifact may compromise a DBS needs to be better understood by careful and systematic investigations. Although LFPs and cardiac dynamics can share some similarities, such as the circadian rhythm, we advocate for careful signal validation to ensure that truly brain‐derived biomarkers are used. That said, even if unintended, it is possible that some patients may still yield positive aDBS effects, even though the stimulation is guided by their heart.

## Author Roles

(1) Research project: A. Conception, B. Organization, C. Execution; (2) Statistical analysis: not applicable; (3) Manuscript preparation: A. Writing of the first draft, B. Review and critique.

M.S.: 1A, 1B, 1C, 3A, 3B

G.T.: 1A, 1B, 1C, 3A, 3B

## Disclosures

**Ethical Compliance Statement:** Written informed consent was obtained from the patient for publication of this case and associated video material. The procedure was conducted in accordance with the guidelines of the local ethics committee at the University Hospital Bern. We confirm that we have read the journal's policy on ethical publication and affirm that this work complies with those standards.

**Funding Sources and Conflicts of Interest:** G.T. has a research agreement with RuneLab; he also receives financial support from Medtronic, Boston, and Spirig, not related to the present work; M.S. receives financial support from Boston Scientific, Medtronic, Zambon, and Bial, not related to the present work.

**Financial Disclosures for the Previous 12 Months:** G.T. receives funding from the Swiss National Science Foundation (project number: PZ00P3_202166) and the Swiss Parkinson Association; M.S. receives funding from Gottfried and Julia Bangerter‐Rhyner‐Stiftung.

## Data Availability

The data that support the findings of this study are available from the corresponding author upon reasonable request.
